# Study and Characterization of Silicon Nitride Optical Waveguide Coupling with a Quartz Tuning Fork for the Development of Integrated Sensing Platforms

**DOI:** 10.3390/s25123663

**Published:** 2025-06-11

**Authors:** Luigi Melchiorre, Ajmal Thottoli, Artem S. Vorobev, Giansergio Menduni, Angelo Sampaolo, Giovanni Magno, Liam O’Faolain, Vincenzo Spagnolo

**Affiliations:** 1 PolySenSe Laboratory, Physics Department, Polytechnic University of Bari, Via G. Amendola 173, 70125 Bari, Italy; l.melchiorre@phd.poliba.it (L.M.); angelo.sampaolo@poliba.it (A.S.); vincenzoluigi.spagnolo@poliba.it (V.S.); 2 nanoPhotonics and Electromagnetics Group, Electrical and Information Engineering Department, Polytechnic University of Bari, Via E. Orabona 4, 70125 Bari, Italy; ajmal.thottoli@poliba.it (A.T.); artem.vorobev@mtu.ie (A.S.V.); giovanni.magno@poliba.it (G.M.); 3 Centre for Advanced Photonics and Process Analysis, Munster Technological University, T12 P928 Cork, Ireland; ms0021925@mtu.ie; 4 Tyndall National Institute, T12 PX46 Cork, Ireland; 5 PolySenSe Innovations S.r.l., Via G. Amendola 173, 70125 Bari, Italy

**Keywords:** LITES, optical gas sensing, photonic integration, piezoelectric resonator, QEPAS, quartz tuning fork, silicon nitride waveguide

## Abstract

**Highlights:**

**What are the main findings?**

- We successfully coupled a silicon nitride waveguide with a custom-designed, low-frequency, and T-shaped QTF, enabling both Quartz-Enhanced Photoacoustic Spectroscopy (QEPAS) and Light-Induced Thermoelastic Spectroscopy (LITES) techniques for sensing.
- We achieved comparable signal-to-noise ratios with QEPAS and LITES when detecting 1.6% water vapor concentration, with performance limited by the output power illuminating the QTF.

**What is the implication of the main finding?**

- Demonstrated the feasibility of integrating photonic components with piezoelectric resonators for portable gas-sensing applications.
- Identified on-chip laser-waveguide integration as a key route to compact sensing platforms.

**Abstract:**

This work demonstrates an ultra-compact optical gas-sensing system, consisting of a pigtailed laser diode emitting at 1392.5 nm for water vapor (H_2_O) detection, a silicon nitride (Si_3_N_4_) optical waveguide to guide the laser light, and a custom-designed, low-frequency, and T-shaped Quartz Tuning Fork (QTF) as the sensitive element. The system employs both Quartz-Enhanced Photoacoustic Spectroscopy (QEPAS) and Light-Induced Thermoelastic Spectroscopy (LITES) techniques for trace gas sensing. A 3.8 mm-wide, S-shaped waveguide path was designed to prevent scattered laser light from directly illuminating the QTF. Both QEPAS and LITES demonstrated comparably low signal-to-noise ratios (SNRs), ranging from 1.6 to 3.2 for a 1.6% indoor H_2_O concentration, primarily owing to the reduced optical power (~300 μW) delivered to the QTF excitation point. These results demonstrate the feasibility of integrating photonic devices and piezoelectric components into portable gas-sensing systems for challenging environments.

## 1. Introduction

Gas-sensing technologies play a vital role across diverse fields, ranging from environmental monitoring and industrial process control to medical diagnostics and security applications [1,2,3,4,5,6,7]. The growing demand for compact, reliable, and highly sensitive detection systems has driven intensive research toward the miniaturization and integration of sensing components [8,9,10,11]. However, traditional approaches, such as conventional spectroscopic methods and electrochemical sensors, rely on bulky equipment or complex free-space optical setups that limit their deployment in real-world, challenging environments [12,13,14,15].

In recent years, optical gas-sensing techniques have proven their advantages in terms of selectivity, response time, and immunity to electromagnetic interference [16,17,18,19,20]. Among these, approaches implementing Quartz Tuning Forks (QTFs) as sensitive elements have gained a significant role in the development of in situ and real-time gas sensors, offering concrete potential for reducing the overall system footprint and enabling large deployment while preserving high reliability. Among the QTF-based approaches, Quartz-Enhanced Photoacoustic Spectroscopy (QEPAS) is the most established technique [19,21,22], relying on the photoacoustic effect, i.e., the generation of acoustic waves by the target gas molecules absorbing modulated laser light and relaxing energy via non-radiative processes [23]. QTFs in QEPAS are employed as sharply resonant acoustic transducers, offering high sensitivity while maintaining a compact system footprint [24,25,26,27,28]. Indeed, this technique demonstrates detection limits in the few parts-per-billion (ppb) range for various gases, down to parts per trillion (ppt) in certain cases, while requiring minimal sample volumes [22,29,30,31,32].

Another approach is Light-Induced Thermoelastic Spectroscopy (LITES), which employs QTFs as photodetectors for gas sensing, exploiting a typical Tunable Diode Laser Absorption Spectroscopy (TDLAS) configuration [33,34,35,36]. The light transmitted through an absorbing gas sample is directly focused onto the surface of the QTF and the portion of optical power released within the resonator generates deformation due to thermoelastic conversion [37]. Thus, a modulation of the residual light beam interacting with the QTF causes periodic heating/cooling, inducing a strain field, which in turn generates a modulation of polarization charges on the tuning fork’s surface, due to quartz piezoelectricity [38]. This second embodiment of QTFs as sensitive elements has demonstrated detection performances comparable to, or even higher than, those of commercially available photoconductive/photovoltaic detectors, and a high and flat spectral responsivity in the 1–10 µm wavelength range [39,40,41,42,43].

For sensing applications, the field of integrated photonics has simultaneously undergone rapid advancement, with Silicon Nitride-On-Insulator (SiNOI) emerging as a promising platform for Near-InfraRed (NIR) applications [44,45,46,47], since it offers a broad transparency window extending from 400 nm to 8 μm and enables applications across a wide spectral range [48,49]. The Si_3_N_4_-based waveguides achieve remarkably low propagation losses, crucial for sensing applications [50,51,52,53], and the high refractive index contrast of silicon nitride facilitates the fabrication of compact device footprints, with efficient light confinement [54]. Furthermore, its CMOS compatibility ensures scalable manufacturing potential, while excellent thermal stability guarantees a reliable operation across varying environmental conditions [55,56,57]. These properties collectively make Si_3_N_4_ particularly suitable for gas-sensing applications, where stable and efficient light delivery is crucial [58,59], creating the potential for building optical systems without the use of discrete optical components, such as lenses, mirrors, and so on.

Nevertheless, significant challenges remain in integrating onto a single chip the fundamental components required for optical gas sensing, i.e., laser source, light/molecule interaction volume, and detector [60]. In this context, optical coupling efficiency remains the most critical parameter, often limiting overall system sensitivity [61,62]. Several studies have explored strategies to integrate the optical sources on various platforms [63] and to design optimized waveguides to enhance light–gas interaction [64]. Considering possible detectors for integrated optical sensing [65], QTFs and Lithium Niobate Tuning Fork (LiNTF) [66] offer high versatility, compact size, and excellent gas-sensing performance, making them promising candidates for integration.

To the best of our knowledge, no previous work has reported a fully or semi-integrated photonic sensing system exploiting on-chip waveguides with such a piezoelectric resonator. The existing literature describes only free-space optical coupling to QTFs [67], all-fiber configurations [68], or 3D-printed acoustic modules [69] but no on-chip waveguide coupling to piezoelectric resonators. While there are reports of integrated photonic gas sensors, these use different detection mechanisms, such as evanescent field absorption or refractive index changes [70], not piezoelectric transduction.

The closest related work, by De Carlo et al., described the modeling and design of a QEPAS sensor in which the laser source and the beam-delivery optics were bonded together and semi-integrated with the QTF [71]. In that configuration, an integrated waveguide couples the laser output to an optical ring resonator located between the QTF prongs, while a pair of mechanical resonators confines and enhances the acoustic standing waves generated by the modulated evanescent optical field. This configuration produced an acoustic wavefront with a pressure comparable to that achieved in free space, on-beam QEPAS. However, the full integration of photonic components with QTFs has yet to be experimentally verified.

In this paper, we demonstrate a novel semi-integrated sensing architecture for QEPAS and LITES that pairs a Si_3_N_4_ optical waveguide with a custom-designed, low-frequency, and T-shaped QTF acting as the detector. The resulting 3.8 mm-wide, S-shaped waveguide path effectively mitigates interference from scattered light, a common issue in integrated sensing platforms [72]. In this proof of concept, we detected water vapor by targeting the absorption line at 7181.16 cm^−1^ [73]. The photonic–piezoelectric components’ integration, along with the use of fiber-based laser beam delivery methods, is a key factor in offering a compact, rugged, and portable sensing system. This development process will also pave the way for the deployment of fully integrated sensors on drones, to empower and push real-time monitoring in dangerous and harsh environments [74,75].

## 2. Semi-Integrated Sensing Architecture

**Figure 1:**
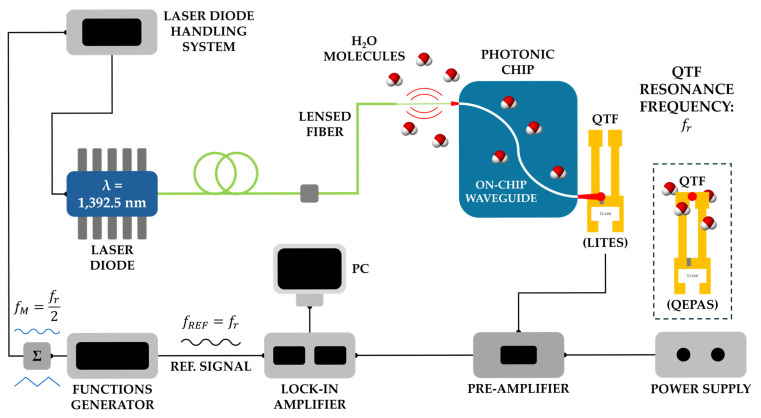
Schematic of the experimental setup for QEPAS and LITES measurements. The QTF position relative to the laser beam spot from the waveguide output port (red dot) differentiates between LITES and QEPAS configurations (dashed box).

Figure 1 presents a schematic of the experimental setup used to perform both QEPAS and LITES measurements.

The experimental setup includes three main components: a laser diode, a Si_3_N_4_ waveguide, and a custom-designed QTF. The optical source is a tunable, single-mode, and pigtailed laser diode (model EP1392-DM-B01-FM, Eblana Photonics Ltd., Dublin, Ireland), emitting at a central wavelength (λ) of 1392.5 nm resonant with the NIR H_2_O absorption feature at 7181.16 cm^−1^ [73]. The laser output was coupled to a tapered lensed fiber (from Highpak Solutions, Suffolk, UK), yielding a focused beam with a spot size of approximately 2.5 μm. The measured laser optical power available at the lensed fiber output to be coupled into the waveguide input was approximately 4 mW.

**Figure 2:**
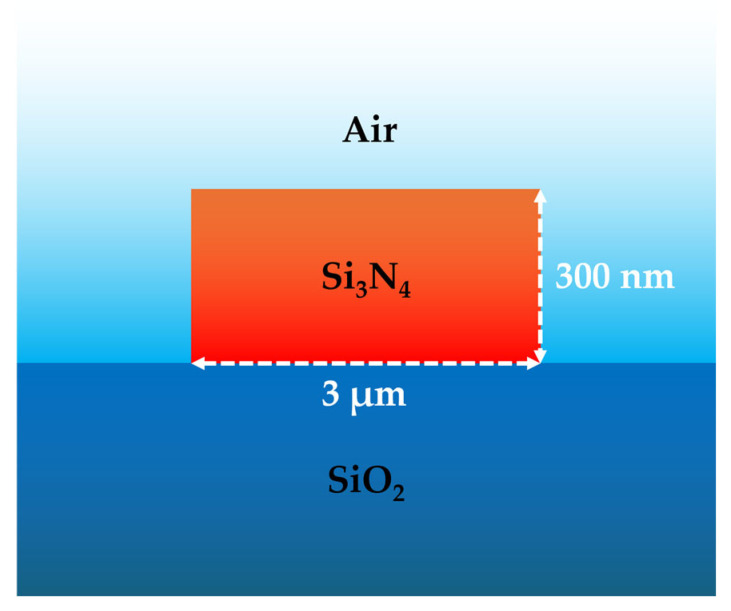
Cross-sectional schematic of the air-clad Si_3_N_4_ optical waveguide structure.

A thermally oxidized silicon wafer with a deposited layer of plasma-enhanced chemical vapor deposition Si_3_N_4_ (300 nm thick) was utilized. The desired optical waveguides layout was then defined on the resist using electron beam lithography. Subsequently, the patterns were transferred to the Si_3_N_4_ layer through an inductively coupled plasma etching process. The cross-section of the waveguide is shown in Figure 2.

**Figure 3:**
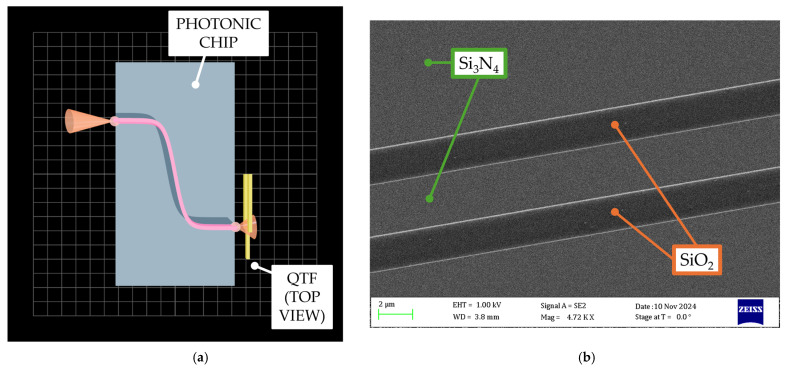
Waveguide details. (**a**) Not-to-scale 3D rendering of the on-chip waveguide topology and (**b**) SEM image of the Si_3_N_4_ waveguide geometry at 4720× magnification (green arrows indicate Si_3_N_4_ layer; orange arrows indicate SiO_2_ one).

The strip waveguide consists of a Si_3_N_4_ (n_Si3N4_ = 2 @λ) core region with a rectangular cross-section, 3 μm in width and 300 nm in height, deposited on a substrate composed of 2 μm-thick silica (n_SiO2_ = 1.45 @λ) [54] over a 525 µm-thick silicon carrier wafer. For LITES detection, the air cladding around the waveguide core along with the polished region of the optical lensed fiber termination provides an absorption path for a direct interaction between the evanescent field and the surrounding gas. Figure 3a,b show more details of the photonic chip.

The overall chip dimensions are 15 mm × 8 mm, and the waveguide has an approximate path length of 9.4 mm, with an input/output offset by 3.8 mm through an S-shaped path designed in the middle section, as shown in Figure 3a. The S-shaped geometry serves both to prevent scattered light from directly illuminating the 1.2 mm-wide QTF side prong [29] and to provide adequate space for potential integrated laser direct bonding to the chip [76]. Figure 3b shows a SEM image taken at 4720× magnification of the waveguide geometry, clearly resolving the silicon nitride waveguide and adjacent silica layer.

The SiNOI platform was chosen for its advantageous optical properties in the tuning range selected for water vapor detection [48], including the propagation loss as low as <0.5 dB/cm potentially achievable using low-temperature fabrication processes directly from foundries [77].

The input laser optical power was P_IN_ ≈ 4 mW and the measured output power illuminating the QTF is approximately P_OUT_ ≈ 300 μW. Instead, by considering only the propagation losses of the employed SiNOI platform (i.e., ~4 dB/cm [78]) and the reflectivity between the air–Si_3_N_4_ interfaces (i.e., ~11.11% [54]), the estimated output power should be P_EST_ ≈ 1.35 mW. The discrepancy between these two values (i.e., a factor of ~4.5) can be attributed to sources such as (i) difficulties in effectively coupling the laser light to the input port of the waveguide, which involves resizing the lensed optical fiber mode to match the waveguide one; (ii) imperfections in the fabrication process; (iii) as well as parasitic reflections within the waveguide structure caused by cleaved facets. The observed laser power discrepancy of ~6.53 dB between P_EST_ and P_OUT_ can be attributed primarily to mode field diameter mismatch and coupling interface losses, common in fiber-to-waveguide photonic systems and capable of reaching values as high as 4 dB/facet, as reported in the literature [79]. By using techniques such as mode adaptors and spot size converters, the optical power illuminating the QTF can be substantially increased [80,81]. The resonance properties of the T-shaped, grooved QTF (model T2-G08 [29]) were retrieved through an electrical characterization of the resonator.

**Figure 4:**
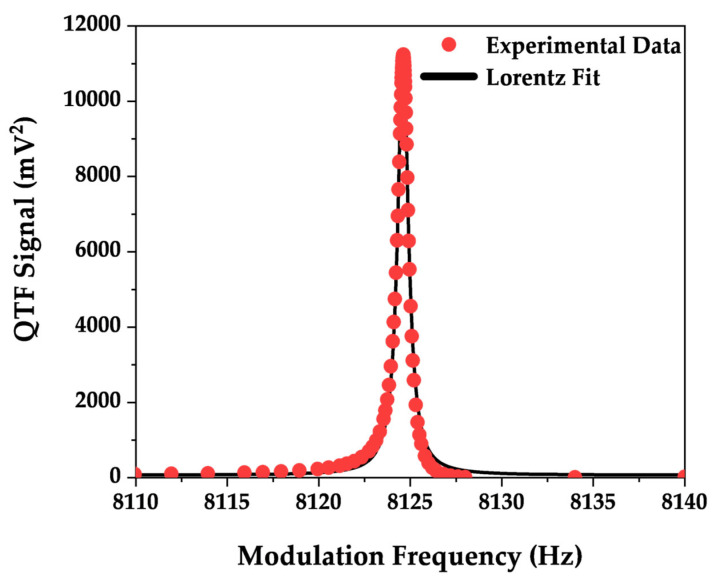
Electrical characterization of the QTF measured under atmospheric pressure. Red dots: experimental data; black line: Lorentzian fit.

Figure 4 shows the electrical response of the QTF (expressed in mV^2^), measured at atmospheric pressure, as a function of the modulation frequency.

The resonance curve shown in Figure 4, fitted with a Lorentzian function, returned a fundamental mode resonance frequency (*f_r_*) of 8124.61 Hz and a quality factor (*Q*) of more than 11,000.

**Figure 5:**
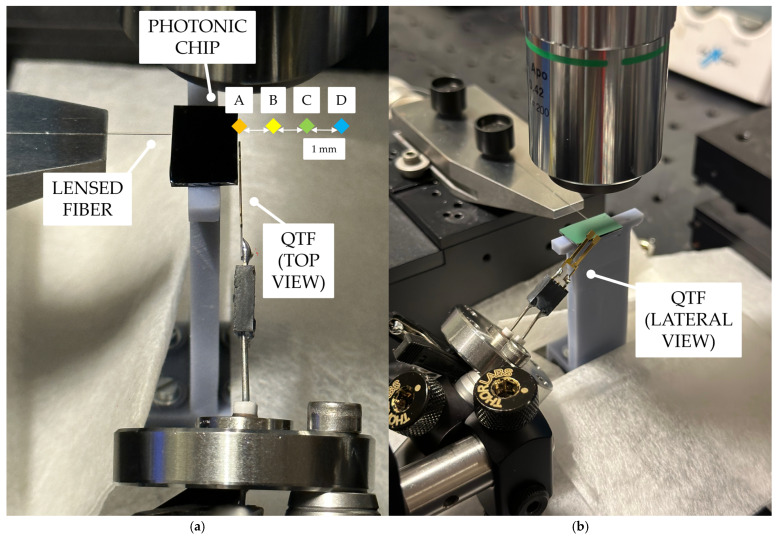
Photographs of the semi-integrated sensing system. (**a**) The lensed optical fiber (left) couples laser light into the on-chip waveguide (center), while the QTF (right) serves as the detector; (**b**) lateral view of the same semi-integrated sensing system.

The QTF and the photonic chip must be positioned close to each other: owing to the high divergence of the laser beam at the waveguide output, stray light can hit the gold electrodes on the QTF surface, leading to a high noise generation [82]. Since the resonance properties could be affected by the presence of the chip in proximity to the QTF surface [83], *f_r_* and *Q* were monitored at different distances between the resonator and the waveguide. Starting from the closest position, i.e., a few tens-of-microns distance, shown in Figure 5a (namely Position A), achieved with the help of a camera and a 20× magnification objective, the QTF was moved away with 1 mm increments to three additional positions (namely Positions B, C, and D), all indicated in Figure 5a, with the help of a translational stage. Moreover, Figure 5b shows a lateral view of the same subject.

No changes in *f_r_* or *Q* were measured at any of the four QTF positions investigated, confirming that the presence of the photonic chip does not affect the QTF resonance properties.

The laser beam was aligned to the on-chip waveguide input port using both a three-axis translation stage (MBT602/M, Thorlabs GmbH, Bergkirchen, Germany)—equipped with a tapered V-groove fiber holder (HFV002, Thorlabs GmbH)—and a Short-Wavelength IR (SWIR) camera (MicronViewer 7290, Electrophysics Corp., Fairfield, NJ, USA) with a 20× objective (MY20X-824, Thorlabs GmbH). The position of the QTF with respect to the laser beam exiting the waveguide output differs between QEPAS and LITES configurations, as shown in the dashed box in Figure 1. Precise positioning of the QTF was achieved using a second three-axis translation stage while monitoring the alignment through the same SWIR camera. All measurements for QEPAS and LITES were repeated at the four above-mentioned QTF distances, at room temperature and under atmospheric pressure (760 Torr) in a humidity-controlled environment. The laser diode temperature and the injected current were controlled using the Laser Diode Handling System (CLD1010, Thorlabs GmbH) shown in Figure 1.

Wavelength Modulation Spectroscopy (WMS) with second-harmonic (*2f*) detection (i.e., *2f*-WMS) was implemented by simultaneously applying the sum of two voltage signals, generated by the Function Generator (AFG31052, Tektronix Inc., Beaverton, OR, USA), to the laser diode controller: a 1 mHz sawtooth wave for scanning the laser tuning range and a sine wave having a frequency of *f_r_*/2 and an amplitude of 85 mVpp for laser injection current modulation. The QTF output signal was amplified using a transimpedance amplifier stage with a feedback resistance of 10 MΩ and then the *f_r_* component was acquired by a lock-in amplifier (7265, PerkinElmer Inc., Shelton, CT, USA). For all measurements, the lock-in integration time constant was set to 0.1 s with a sampling time of 0.3 s. A DAQ card (USB-6002, National Instruments Corp., Austin, TX, USA) allowed data acquisition and storage on a Personal Computer (PC).

## 3. QEPAS and LITES Results

To evaluate the performance of our semi-integrated sensing architecture, both QEPAS and LITES techniques were employed to detect water vapor molecules in a laboratory environment. For both sets of measurements, the optimal position of the QTF with respect to the waveguide output was determined by maximizing the H_2_O QEPAS (and LITES) signal.

**Figure 6:**
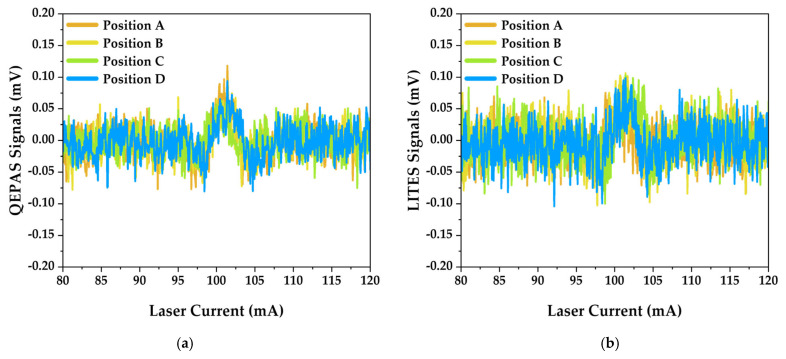
*2f*-WMS spectra over the laser tuning range at four different QTF positions for (**a**) QEPAS and (**b**) LITES measurements. Position A represents the closest distance between the QTF and waveguide output, with subsequent positions (B, C, and D) spaced at 1 mm increments.

Figure 6a and Figure 6b show the QEPAS and LITES spectra, respectively, for indoor H_2_O detection.

For both QEPAS and LITES sets of measurements and for all the QTF positions investigated, a resolved signal corresponding to H_2_O detection was retrieved around 101.41 mA, which corresponds to the targeted H_2_O absorption feature localized at 7181.16 cm^−1^ [73]. The resolved signals were obtained with an output laser optical power illuminating the QTF of ~300 μW.

The measured signals correspond to a 1.6% absolute humidity of the indoor controlled environment, i.e., with a fixed temperature of 15 °C kept constant by an in-house A/C cooling system and with a gas pressure of 760 Torr. The constant value of water concentration was monitored through a humidity logger (TSP01, Thorlabs GmbH), which recorded relative humidity fluctuations on the order of a few percent during the whole set of measurements.

A noise analysis was performed by calculating the standard deviation of the QEPAS (and LITES) signals in an absorption feature-free region within the laser tuning range (i.e., ranging from a 40 mA to 60 mA laser injection current). In Table 1, the noise levels for both techniques at different positions are reported.

The slightly lower noise levels observed in QEPAS measurements suggest that the light at the output of the waveguide can pass through the resonator prongs without hitting them, while it is difficult to focus light onto a precise spot of the QTF surface for LITES detection [33] without the help of a focusing lens. Moreover, in QEPAS, the photoacoustic effect occurring in the gas molecules localized between the prongs minimizes direct optical interaction with the QTF surface. Conversely, LITES requires the direct illumination of the QTF surface for thermoelastic effect, making it more susceptible to stray light and thermal noise. However, the laser beam must not hit the gold electrodes since, otherwise, a large, undesirable, non-zero background arises due to the photo-thermal contribution, limiting the sensor detection sensitivity [26].

### Experimental Data Fitting

As the peak signals for both spectroscopic configurations were barely recognizable above the background noise and to provide meaningful estimations of the signal-to-noise ratio (SNR), a data fitting procedure using the second derivative of a Voigt profile [84] was implemented to properly determine the peak values at the four positions for both QEPAS and LITES. This, in turn, enabled a proper comparison of SNRs.

The Voigt profile, *V*(*x*;*σ*,*γ*), is expressed as:

(1) $$V\left(x;\sigma ,\gamma \right)\equiv \int_{-\infty }^{+\infty }G\left(x^{\prime };\sigma \right)L\left(x-x^{\prime };\gamma \right)dx^{\prime },$$

where *x* is the shift from the absorption line center, and *G*(*x*;*σ*) is the centered Gaussian distribution:

(2) $$G\left(x;\sigma \right)\equiv \frac{e^{-\frac{x^{2}}{2\sigma^{2}}}}{\sqrt{2\pi }\sigma },$$

where *σ* is the Gaussian width parameter (related to Doppler broadening), and *L*(*x*;*γ*) is the centered Lorentzian distribution:

(3) $$L\left(x;\gamma \right)\equiv \frac{\gamma }{\pi \left(\gamma^{2}+x^{2}\right)},$$

where *γ* is the Lorentzian width parameter (related to pressure broadening). In *2f*-WMS, when the laser wavelength is modulated at frequency *ω*, the detected second harmonic signal can be modeled as proportional to the second derivative of the absorption profile [85]:

(4) $$S_{2f}\propto \frac{d^{2}}{dx^{2}}\left[V(x;\sigma ,\gamma )\right].$$

The second derivative Voigt fitting function is implemented in a Python code (Python 3.11) as:

(5) $$V_{2f-FIT}\left(x\right)=-a\cdot \frac{d^{2}}{dx^{2}}\left[V\left(x-x_{0},\sigma ,\gamma \right)\right]+y_{0}$$

where *a* is the amplitude scaling factor; *x*_0_ is the position of the peak in the scan; *σ* is the Gaussian width parameter; *γ* is the Lorentzian width parameter; and *y*_0_ is the baseline offset. The minus sign in Equation (5) was introduced to account for the detection phase in the lock-in detection.

**Figure 7:**
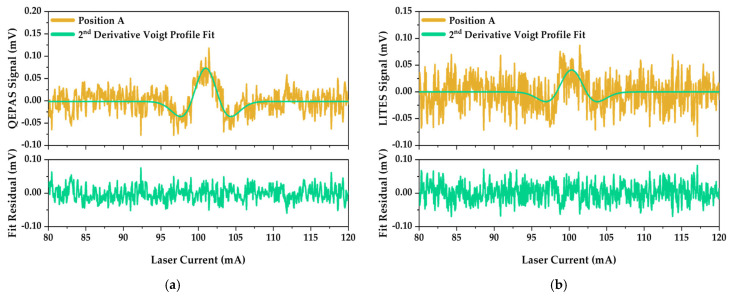
Second-derivative Voigt profile fitting of the (**a**) QEPAS and (**b**) LITES signals at QTF Position A. Green curves represent optimized Voigt profile fitting the experimental data (orange curves). Lower panels report the fit residual for both techniques.

Figure 7a and Figure 7b show the fitted spectra for representative Position A for both QEPAS and LITES techniques, respectively, along with the corresponding raw signal as a function of the laser current.

The fitted signals and the corresponding residuals shown in Figure 7 demonstrate the effectiveness of this fitting procedure.

Peak values at different QTF positions (A through D) were extracted from the fitted signals for both QEPAS and LITES measurements and the resulting SNR values are presented in Table 2.

The SNR values presented in Table 2 demonstrate that both QEPAS and LITES techniques achieve comparable detection performances. The highest SNR was obtained with QEPAS at Position A (3.2), while LITES showed its best performance at Position B (2.8). By taking into account these results and considering an H_2_O indoor concentration of 1.6%, a Minimum Detection Limit (MDL) of 0.5% and of 0.6% is achievable with QEPAS and LITES techniques, respectively. Nevertheless, both techniques maintained an effective detection capability across the entire tested distance range, with SNRs consistently above 1.5 at an optical power of ~300 μW, thus showing both good mechanical and optical stability. The generally lower SNRs observed in the LITES measurements align with the higher noise levels previously reported in Table 1 and discussed alongside it.

## 4. Discussion

The experimental results demonstrate the successful coupling between Si_3_N_4_ waveguide technology and a custom-designed QTF, leveraging both QEPAS and LITES detection techniques for gas-sensing applications. This semi-integrated architecture represents a significant step toward miniaturized optical gas sensors, particularly for deployment in challenging environments where traditional approaches may be impractical.

The comparable SNR values obtained for both QEPAS (1.9–3.2) and LITES (1.6–2.8) techniques indicate that either method can be effectively implemented with the reported semi-integrated platform.

The slight performance advantage observed in QEPAS measurements, particularly at QTF Position A (SNR = 3.2), is partly due to lower noise levels, thus indicating that it is easier to align the beam at the output of the waveguide between the QTF prongs.

For the LITES case, the small light–molecules interaction volume considered, localized around the polished region of the optical lensed fiber and around the waveguide core-cladding interface, limits performance.

However, the consistent detection capability across all positions (up to 3 mm from the waveguide output) demonstrates the robustness of both techniques and provides flexibility in sensor design and implementation.

The integration of the Si_3_N_4_ optical waveguide proves to be particularly advantageous, with the S-shaped design effectively preventing scattered light interference while maintaining acceptable optical losses. The measured output laser optical power at the QTF surface of ~300 μW, although relatively low, still enabled successful gas detection. This suggests potential for further optimization of the coupling efficiency and/or laser optical power to enhance sensor performance.

## 5. Conclusions

This work demonstrates a proof of concept for a novel, semi-integrated optical gas-sensing architecture that combines a Si_3_N_4_ waveguide with a custom-designed, low-frequency, and T-shaped QTF. The system operates with both QEPAS and LITES using minimal optical power (~300 μW) and achieves a comparable performance (SNRs of 1.6–3.2) for water vapor detection in a laboratory environment, confirming its versatility and robustness. The integration of photonic and piezoelectric components represents a significant step toward miniaturized gas sensors.

These low SNRs stem primarily from the limited laser optical power available for sensing. Future developments can increase the available power by optimizing mode matching [78], refining integrated coupling structures [71], or integrating the laser source directly on-chip [76,86]. Furthermore, we plan to implement and test an optical lens that collects the beam at the waveguide output and focuses it onto the detector to lower noise [87]. Future work should also include comprehensive repeatability and long-term stability studies to fully characterize the practical reliability of the sensor for deployment applications. Compatibility with both QEPAS and LITES establishes the foundation for multimodal detection methodologies, which can enhance measurement reliability through complementary sensing and redundant signal validation [40]. The compact nature of this architecture also opens the way to multiplexed sensing via integrating multiple waveguide and laser sources on a single chip [88].

## Figures and Tables

**Table 1:** Noise levels for both QEPAS and LITES techniques at different QTF positions (A through D).

Techniques	QTF Positions			
	A	B	C	D
QEPAS	0.023 mV	0.019 mV	0.022 mV	0.019 mV
LITES	0.026 mV	0.027 mV	0.026 mV	0.028 mV

**Table 2:** SNRs calculated using peak values extracted from fitted signals for both QEPAS and LITES techniques at different QTF positions (A through D).

Techniques	QTF Positions			
	A	B	C	D
QEPAS	3.2	2.7	1.9	2.9
LITES	1.6	2.8	2.5	2

## Data Availability

The raw data supporting the conclusions of this article will be made available by the authors on request.

## Abbreviations

The following abbreviations are used in this manuscript:

*2f*	Second harmonic
CMOS	Complementary Metal-Oxide Semiconductor
DAQ	Data acquisition
*f_r_*	Resonance frequency
H_2_O	Water vapor
IR	Infrared
LiNTF	Lithium Niobate Tuning Fork
LITES	Light-Induced Thermoelastic Spectroscopy
MDL	Minimum Detection Limit
NIR	Near-IR
PC	Personal Computer
*Q*	Quality factor
QEPAS	Quartz-Enhanced Photoacoustic Spectroscopy
QTF	Quartz Tuning Fork
SEM	Scanning Electron Microscope
Si_3_N_4_	Silicon nitride
SiNOI	Silicon Nitride-On-Insulator
SiO_2_	Silicon dioxide/silica
SNR	Signal-to-noise ratio
SWIR	Short-Wavelength IR
TDLAS	Tunable Diode Laser Absorption Spectroscopy
WMS	Wavelength Modulation Spectroscopy
